# The anti-obesity effects of polyphenols: a comprehensive review of molecular mechanisms and signal pathways in regulating adipocytes

**DOI:** 10.3389/fnut.2024.1393575

**Published:** 2024-10-30

**Authors:** Lan He, Zhan Su, Shuangshuang Wang

**Affiliations:** ^1^Department of Cardiology, The First People’s Hospital of Wenling, Taizhou University Affiliated Wenling Hospital, Zhejiang, China; ^2^College of Bioscience and Biotechnology, Hunan Agricultural University, Changsha, Hunan, China; ^3^Key Laboratory of Precision Medicine for Atherosclerotic Diseases of Zhejiang Province, Affiliated First Hospital of Ningbo University, Ningbo, China

**Keywords:** polyphenol, obesity, adipose tissue, antioxidant effect, gut microbiota, inflammatory reaction, signal pathways

## Abstract

Excess weight gain is a growing concern worldwide, fueled by increased consumption of calorie-dense foods and more sedentary lifestyles. Obesity in China is also becoming increasingly problematic, developing into a major public health concern. Obesity not only increases the risk of associated disease but also imposes a burden on health care systems, and it is thus imperative that an effective intervention approach be identified. Recent studies have demonstrated that the polyphenol-rich Mediterranean diet has considerable potential in this regard. Polyphenols can inhibit the production of adipocytes and reduce adverse reactions, such as inflammation, insulin resistance, and gut microflora imbalance. In this review, we examine four polyphenols (curcumin, ellagic acid, ferulic acid, and quercetin) in terms of their potential as interventions targeting obesity. The mechanisms that help promote adipocyte browning, increase thermogenic factors, increase thermogenesis, and regulate adipocyte differentiation are summarized, and key signaling pathways, including PPARγ, C/EBP-, and others, are reviewed.

## 1 Introduction

**Figure 1:**
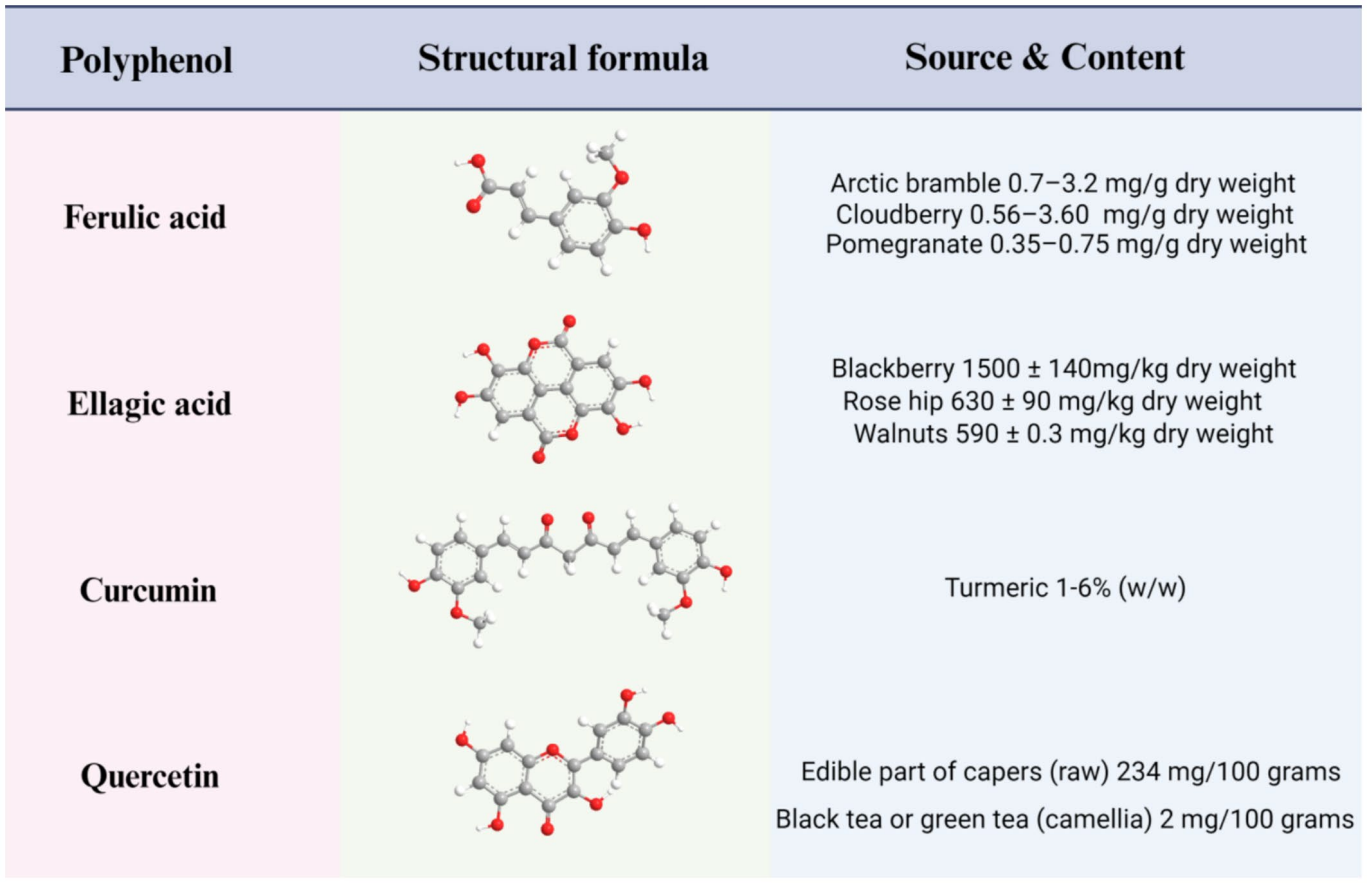
Structures, main sources, and contents of four polyphenols: ferulic acid, ellagic acid, curcumin, and quercetin.

People’s standard of living has risen consistently since 1980, and long-term sedentary lifestyles have proliferated in tandem (1, 2). Accordingly, the worldwide incidence of overweight has seen a twofold increase, with roughly one-third of the global population now classified as either obese or overweight (3, 4). Obesity has reached epidemic levels and is acknowledged as a serious medical condition worldwide. Mirroring trends witnessed around the globe; obesity has become a significant public health concern in China (5). Obesity, a chronic disease, has diverse causes, including genetic factors, living environment, psychological status, and social background (6). Body Mass Index (BMI), a prevalent metric obtained by dividing an individual’s weight in kilograms by their height squared in meters, serving as a broad gauge of weight health, is commonly used to determine weight status (7). Generally, a BMI of less than 18.5 is considered to be underweight; the optimal body weight lies between 18.5 and 24.9; and a BMI between 25 and 29.9 is considered overweight. Obesity is defined as a BMI of greater than or equal to 30 (7–9). The BMI metric fundamentally rests on the principle of energy balance, reflecting the discrepancy between an individual’s energy intake from food and the energy they expend through engagement in daily activities (10). An excess of energy intake over energy expenditure is typically regarded as signifying obesity (11). Recently, shifts in dietary habits, decreased physical exercise, and disruptions in hormonal balance due to irregular sleep patterns have collectively contributed to a yearly rise in obesity cases, and a solution to this problem is urgently required. At present, three main approaches to promoting weight loss are recognized. The first is a combination of high-intensity exercise and low-calorie diet. However, compliance with this method is typically poor, and the results are often unsatisfactory; The second approach is surgical intervention, such as sleeve gastrectomy (12), now the world’s most prevalent surgery for treating severe obesity. Although its efficacy has been confirmed, preexisting or new gastroesophageal reflux disease and its potential complications, such as peptic esophagitis, Barrett’s esophagus and—in the long-term—esophageal adenocarcinoma, have emerged as a major drawback to this method (13). The third approach is pharmacological treatment, but most of the associated drugs entail adverse side effects, particularly affecting the gastrointestinal (GI) tract, liver, and blood vessels (8, 14, 15). Owing to the limitations of the above three measures, a more reasonable, healthy, and economic weight loss method is a desideratum. Recent research has affirmed that adherence to a Mediterranean diet has the potential to significantly reduce obesity, metabolic syndrome, and associated chronic disease risks, echoing the findings of previous studies (16–18). In an 18-month randomized controlled trial of a dietary polyphenol intervention, it was found that a polyphenol-rich Mediterranean diet promoted visceral fat loss (19). Recent studies have shown that the Mediterranean diet significantly reduces the risk of obesity, metabolic syndrome and related chronic diseases, and its higher dietary polyphenol content may be one of the reasons (20). Moreover, these polyphenols exhibit multiple benefits, including antioxidant and anti-inflammatory properties, in addition to aiding in blood sugar control and obesity prevention (21–23). Therefore, the scientific community regards polyphenols as having significant potential as a means of combating the obesity problem. Current taxonomic evidence indicates that polyphenols may be classified according to their origin, function, or structure. The latest classification divides them into two main categories: flavonoids and non-flavonoids (24, 25). Within the broad category of non-flavonoid polyphenols, several subcategories with potential health benefits have been identified. Among these, phenolic acids have emerged as preeminent, with other unclassified polyphenols also included. The representative substances of flavonoids are quercetin and Epigallocatechin-3-gallate (EGCG). EGCG’s mechanisms in the intervention of obesity have gradually come to be appreciated and include the regulation of the STAT1/SLC7A11 pathway, gut microbiota, leptin, and other mechanisms (26, 27). As such, we will not discuss EGCG in the present article but will focus on quercetin, as far as flavonoids are concerned. Among the non-flavonoids, we shall examine ferulic and ellagic acids as key phenolic acids in addition to curcumin as an unclassified polyphenol. The four polyphenols mentioned herein include curcumin (C_21_H_2_0O_6_); quercetin (C_15_H_10_O_7_); ferulic acid 3-methoxy-4-hydroxycinnamic acid with chemical formula C_10_H_10_O_4_; and ellagic acid 2, 3, 7, 8-tetrahydroxy benzopyrano5, 4, 3 a cdebenzopyran – 5, 10 a dione, with chemical structural formula C_14_H_6_O_8_. Figure 1 details the main sources of the natural polyphenols and their contents (28–32). In this review, we shall discuss these four polyphenols’ anti-obesity mechanisms.

## 2 Obesity

### 2.1 Fat cells

Adipocytes are central to fat tissue and are vital for maintaining lipid metabolism and managing energy effectively (33). Adipocytes are classified into two main types according to their function: white adipose tissue (WAT) and brown adipose tissue (BAT) (6). WAT primarily holds extra calories as triglycerides. Not only act as a reservoir for energy storage and utilization but it is also the body’s largest endocrine organ, secreting fat hormones and cytokines. Fatty acids participate in various metabolic and physiological signal cascades, regulating energy production and metabolic processes, such as insulin signal transduction, glucose uptake, and fatty acid oxidation (34), while cytokines regulate inflammation and inflammation resolution as well as adaptive and repair angiogenesis (35). BAT functions as a heat source as it contains a large number of mitochondria, which are heavily involved in energy expenditure and heat production. BAT has a unique structure, containing multiple lipid droplet units and a large number of mitochondria that contain a key protein component known as uncoupling protein 1 (UCP1). As a key protein located in the inner mitochondrial membrane, UCP1 is known to play a crucial role in regulating heat production, which is non-mitochondrial respiration caused by the synthesis of triphosphate (36). Recent studies have demonstrated that beige fat, a transitional type between WAT and BAT, expresses high levels of UCP1 and can dynamically react to stimuli such as exercise, cold, or certain hormones to boost thermogenesis (37). Under normal conditions, beige adipocytes have similar molecular marker expression characteristics to WAT; after trans differentiation, however, their expression patterns resemble those of BAT (38). Furthermore, adipose tissue comprises various cell types, including macrophages, fibroblasts, pre-adipocytes, and mature adipocytes. In addition to mature adipocytes and their progenitor cells, endothelial cells and various immune cell types are also included, which together are responsible for the development and function of adipose tissue (39). Extensive research has emphasized the pivotal role that adipose tissue conditions play in the development of several metabolic disorders, ranging from obesity to its associated complications (40).

### 2.2 Complications of abnormal adipose tissue due to obesity

#### 2.2.1 Dysregulation of gut microflora

Studies have shown that obesity may cause dysregulation of gut microflora (41). This is due primarily to the change in the proportion of microbial species and the loss of overall intestinal microbial diversity, which leads to intestinal microbial imbalance.

Mouse model studies have shown that Bacteroidetes and Firmicutes typically dominate the gut microbiome (comprising 92.6%). However, researchers observed a 50% decrease in Bacteroidetes alongside a proportional rise in Firmicutes in genetically obese mice compared to lean mice (42). A study involving a human model of obese children obtained the same conclusion, with weight loss following 1 year of diet found to increase Bacteroidetes and decrease Firmicutes (43). However, some studies reported the opposite ratio for human participants (44). Nonetheless, it may be stated with certainty that obesity can cause an imbalance in intestinal microbes. However, further studies are necessary to determine which species are preferable.

Moreover, a growing body of research points to notable differences in the composition and diversity of gut microflora between obese individuals and healthy individuals (45, 46). Obese individuals exhibit a considerable decline in gut bacterial diversity compared to control groups (47). Following PCR analysis, *Million et al.* found that obese individuals showed an increase in *Lactobacillus reuteri* and a decrease in *Methanobrevibacter smithii* compared to overweight, lean, and anorexic subjects. Notably, this study also reported, for the first time, a negative correlation between *E. coli* levels and BMI (48). Concurrently, *Murga-Garrido et al.* research revealed that children with normal weight exhibited heightened proportions of *Bacteroides rodentium*, *B. intestinalis*, *B. eggerthii*, and *Methanobrevibacter smithii* in their gut microbiota. By contrast, overweight and obese children exhibited increased levels of *Eubacterium* species and *Roseburia* species (46).

#### 2.2.2 Inflammation

Dysregulation of gut microflora can further aggravate the occurrence of enteritis because when gut microbes are dysregulated, lipopolysaccharide (LPS) content tends to increase (49, 50). Owing to its affinity, LPS’s lipid A component can either bind directly to the intestinal lining or traverse the gut barrier aided by chylomicron transport (50). LPS crosses the intestinal barrier and interacts with LPS-binding proteins to activate CD14 receptors. This activation facilitates the assembly of a complex that docks onto Toll-like receptor 4 on macrophage and adipose tissue surfaces, triggering a signaling cascade. This signaling pathway essentially stimulates the expression of various genes coding for inflammatory proteins, notably including nuclear κB factor (NF-κB) and activator protein 1, among others (51), thus inducing enteritis. Obesity can not only mediate inflammation by regulating gut microbiota, but also directly induce inflammation.

The current scientific consensus that obesity manifests as a persistent, low-level inflammatory state reveals a profound link between adipose tissue and inflammatory processes. The current view is that energy-dense diets are the underlying source of the increased amount and volume of adipose tissue. It has been shown that hypoxia modulates the production of several adipokines associated with inflammation. Hypoxia also induces an inflammatory response in macrophages, which may be a key factor in adipose tissue dysfunction. On the other hand, hypoxia induces inflammation and adipocyte dysfunction by affecting WAT, as well as immune cell infiltration of interstitial vessels (52). Locally and throughout the body, adipocytes secrete inflammatory factors, thereby disrupting the function of their own and distant organs (53). Of course, as well as secreting pro-inflammatory cytokines, adipocytes also make anti-inflammatory cytokines. However, in the presence of overweight, the production and release of such anti-inflammatory components appear to show a downward trend, which is due to obesity’s tendency to enhance the action of pro-inflammatory adipokines in the body, thus disrupting the delicate balance between anti-inflammatory and pro-inflammatory responses (35). This, in turn, promotes obesity.

#### 2.2.3 Insulin resistance

Inflammation also affects insulin sensitivity, and abundant evidence suggests that inflammation is directly related to insulin resistance. Normally, NF-κB and JNK, as well as the inflammatory factor interleukin-6 (IL-6), mediate insulin resistance (54, 55). The latter is pivotal in fine-tuning the insulin signaling pathway, both suppressing insulin receptor substrate 1 production and markedly decreasing cytokine signaling suppressor activity (56). Cytokine signaling inhibitors, which are negative feedback regulatory molecules in the cytokine signaling pathway, are a class of inflammatory mediators that promote obesity and induce insulin resistance (57).

Based on the above, the reduction of obesity may be approached from two aspects: the regulation of fat cells and the mitigation of complications caused by abnormal fat cells.

## 3 The regulatory mechanism of common polyphenols on obesity

In accordance with the polyphenol’s classification, we have summarized the mechanisms by which four representative substances of polyphenols regulate obesity. These include quercetin, a flavonoid; ferulic acid, a phenolic acid, ellagic acid, which is found in Radix Astragali; and curcumin, categorized as another type of polyphenol. However, few studies have examined the effects of lignans and tannins on obesity, and many potential mechanisms have not been discovered and are thus not mentioned in this paper. The above-mentioned four substances function primarily by increasing heat production, inhibiting fat production, regulating fat metabolism, reducing inflammation, improving insulin sensitivity, and regulating the intestinal tract.

### 3.1 Increase thermogenesis

#### 3.1.1 Induce WAT conversion to beige adipose tissue

Beige fat, characterized by UCP1 expression, emerges from the browning of WAT in response to specific triggers, such as exercise, cold exposure, or particular hormones (58). The specific hormone here refers to thyroid hormones (59). While beige fat cells initially share molecular markers with WAT, they adopt characteristics that resemble those of BAT following transdifferentiating (38). As such, they can also perform thermogenic functions. Thus, increasing the amount of beige fat tissue can promote thermogenesis. In a study by *Wang et al.*, the experimental group added high-dose ellagic acid (HF + HD ellagic acid) to rats following a high-fat diet; *N* = 8; 30 mg/kg/day. The experiment involved a high-fat diet supplemented with a low dosage of ellagic acid, labeled as the HF + LD ellagic acid group, consisting of 8 groups, for which the administered ellagic acid dose was 10 milligrams per kilogram of body weight daily. The study observed that the weight loss in mice may be due to ellagic acid’s ability to inhibit WAT maintenance genes, promote WAT browning, and significantly reduce the mRNA expression of Zfp423 and Aldh1a1 (60). The action of ellagic acid is a decisive factor in the plastic process of WAT. Notably, ellagic acid was found to induce an increase in the expression of beige markers in WAT, such as differentiation cluster 137 and transmembrane protein 26 (60, 61). Ellagic acid facilitates the conversion of WAT into beige adipose tissue, characterized by heightened energy expenditure capabilities. This promotion is evidenced by elevated levels of specific markers, thereby accelerating the browning of WAT. This plays a central role in unraveling the mechanism of reversible WAT transition.

#### 3.1.2 Increase the activity of BAT

Accumulating evidence attests that genetically modified organisms have significant relevance for and potential effects on energy metabolism. Several studies have shown that approximately one-third of blood metabolites in mammals may be derived from genetically modified organisms, of which bile acids (BAs) is a prominent example (62). The initial synthesis of BAs occurs in the liver, after which they further undergo a biotransformation process under the action of gut microbes (63). They not only exist in a variety of tissues as key signaling molecules but through interaction with specific BAs receptors, they can activate the related signal transduction pathways and exert far-reaching regulatory effects on the overall host metabolic activities (62, 64). Specifically, BAs boost BAT activity by interacting with Takeda G-protein-coupled receptor 5 receptors (65). Curcumin, as a polyphenol, is proven to increase energy consumption.

In a recent study, *Han et al.* investigated whether curcumin’s energy balance-enhancing effect was influenced by genetic modifications (66). They performed an experiment involving UCP1 and G-protein-coupled membrane receptor 5 knockout mice, feeding them a high-fat diet (HFD) while administering curcumin at 100 mg/kg. Their results indicated that curcumin-treated mice experienced notably less weight gain than their counterparts on the same diet without curcumin supplementation. Moreover, these curcumin-treated mice showed enhanced cold tolerance due to activation of adaptive thermogenic mechanisms *in vivo*. In addition, it was found that the anti-obesity effect of curcumin was affected when UCP1 was knocked out. In-depth analyses, including 16S ribosomal DNA sequencing, revealed that curcumin induced gut microbial (GM) community reconfiguration in these specific mouse models. Curcumin has been found to regulate BAs metabolism, and increases level of the G protein coupled receptor 5 two strongest ligand deoxycholic acid and lithocholic acid so as to increase the activity of BAT (66).

#### 3.1.3 Increase the protein factors that regulate BAT thermogenesis

**Figure 2:**
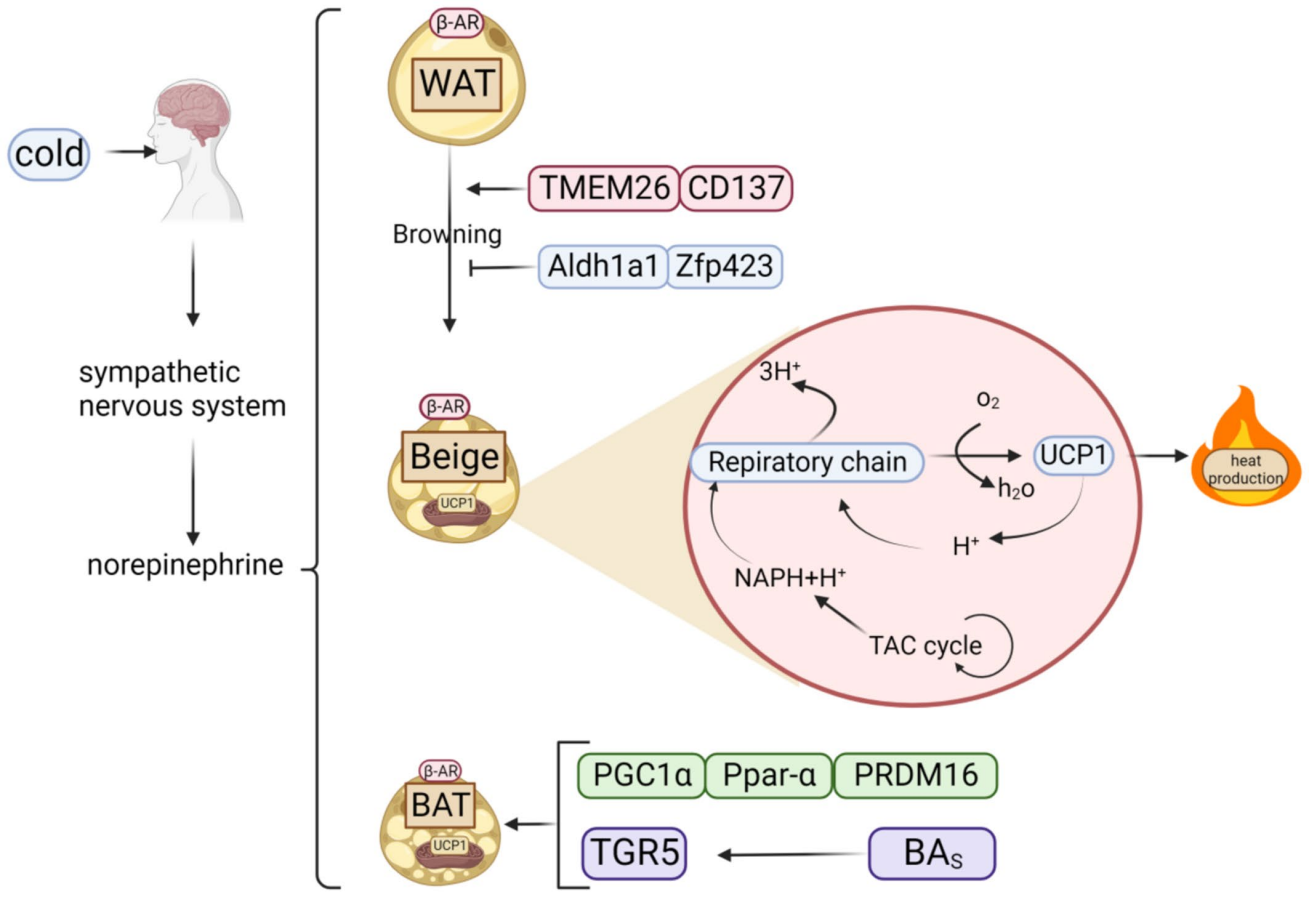
Classification of fats and possible thermogenic mechanisms. When cold stimulates the brain, it triggers the sympathetic nervous system to release norepinephrine, which acts on *β*-AR to activate three types of adipose tissue: WAT, BAT, and beige adipose tissue, created by browning of WAT. Beige adipose tissue can also generate heat via respiratory chain because it contains UCP1, a key thermogenic protein of BAT.

BAT is vital for thermogenesis, largely through its interaction with heat-producing proteins. Effective regulation of these proteins is key to boosting heat generation. Polyphenols can inhibit obesity by increasing protein factor, which regulates the heat production of BAT (Figure 2).

*Wang et al.* founded that high dose is added in the high-fat feed ellagic acid (30 mg/kg/day) and low dose of ellagic acid (10 mg/kg/day) of mice, compared with the mice did not add the ellagic acid, increase mRNA expression of the BAT markers UCP1, PRDM16, PGC1*α* and Ppar-α (67). A recent study by KJ Kim revealed that ellagic acid supplementation in mice elevated (carnitine palmitoyltransferase-1) CPT1, UCP1, and PGC1α protein levels in a dose-dependent manner—1 mg/kg, 2 mg/kg, and 4 mg/kg, respectively, suggesting that ellagic acid’s anti-obesity potential stems from its energy-boosting properties (67). However, it is important to know that each polyphenol intervenes in obesity by different mechanisms and has different effects on obesity-related pathways, e.g., curcumin and other polyphenols are differently effective for affecting NAD^+^ (68), and therefore should be applied on a case-by-case basis.

*Choi et al.* performed an experiment wherein they randomly divided a cohort of 8-week-old male C57BL/6 mice into two separate control groups. One group received a high-caloric intake alone, whereas the other group was given a high-calorie regimen enriched with 0.05% (w/w) quercetin of no less than 95% purity, designated as the HFD + 0.05% Que. group. The researchers’ intention with this grouping strategy was to elucidate quercetin’s potential impact on mouse obesity. The outcomes demonstrated that incorporating quercetin in the diet elevated Ucp1 levels and amplified the mRNA expression of thermogenesis-related genes in HFD-induced obese mice (69).

### 3.2 Inhibit fat formation

The adipocyte formation process begins with mesenchymal cells, which undergo a series of differentiation steps to first transform into precursor cells and subsequently further mature into fully functional adipocytes. Overregulation of fat production can lead to excessive accumulation of fat cells, which in turn leads to obesity (70). Adipocyte development may be divided into several distinct stages, including growth arrest, mitotic proliferation, early differentiation, and, finally, mature differentiation (71). Any stage of adipogenesis is subject to interruption by multiple factors. Polyphenols can inhibit the formation of fat through signal pathway regulation and transcription factor regulation thus exerting an anti-obesity effect.

#### 3.2.1 Signal path regulation

The retinoblastoma (Rb) protein signaling pathway plays a significant role during the initial stages of fat cell development. Lipogenic hormones induce Rb phosphorylation through ring-dependent kinase pathways, leading to the separation of Rb/e2f complex, while e2f promotes cell cycle progression to the S phase. Both ellagic acid and curcumin significantly inhibit hormone-induced lipogenesis in a dose-dependent manner. *Wang et al.* examined the maturation of 3 T3–L1 pre-adipocytes by subjecting them to distinct ellagic acid levels (0, 10, 15, 20 μM) in the culture medium, renewed every 48 h over an 8-day period. The results indicated that with the increase of ellagic acid concentration in the culture medium, the accumulation of neutral lipids in 3 T3–L1 cells exhibited a significant and dose-dependent decrease. Treatment, particularly when measured at 20 μM ellagic acid, significantly reduced adipogenesis (72). *Asma Ejaz et al.* subjected 3 T3–L1 cells to varying concentrations of curcumin (0, 5, 10, 20 μmol/L) for a duration of 24 h with the objective of understanding the influence of diverse curcumin dosages on these cells. Their findings yielded similar conclusions (73). The underlying mechanisms of curcumin and ellagic acid inhibition concentration of 3 T3 – L1 may be by reducing Rb phosphorylation, blocking the G1/S phase shift, inhibiting the 3 T3–L1 terminal differentiation of fat cells and lipid accumulation, thus inhibiting fat cells to form (72).

Throughout the progression of human cells from precursors to fully developed adipocytes, an amplification occurs in the activity of P38 mitogen-activated protein kinase (P38 MAPK). Pharmacological interference with P38 MAPK’s function and suppression of its activity in these cells notably reduces triglyceride buildup and effectively diminishes the expression of PPAR, a marker that is intimately tied to adipocyte differentiation. This intervention decelerates or impedes their transition into mature fat cells, suggesting that P38 MAPK is instrumental in priming human primary cells for differentiation. A separate study demonstrated that using a tailored P38 MAPK inhibitor effectively hindered the adipogenic conversion of 3 T3–L1 cells. Treatment with P38 inhibitors reduced cytokine enhancer-binding polypeptide *β* (CEBP-β) phosphorylation *in vivo* and correspondingly reduced PPARγ phosphorylated expression. This suggests that CEBP-β may be a target of P38 MAPK in the regulation of adipogenesis and that P38 MAPK’s enhanced activity promotes the differentiation of 3 T3–L1 cells during the early differentiation stage of adipocytes. We found that curcumin (5 mΜ)-pretreated human THP1 macrophages secreted by Interleukin-1β(IL-1β) intestinal epithelial cells (IEC), PPARγ differentiation, or mouse peritoneal macrophages induced by thioglycolate for 48 h and then pretreated overnight with LPS (1 μg/mL) reduced IL-1β-induced p38 MAPK activation in IEC (74). The P38 MAPK signaling pathway is involved in the secretion of leptin, thereby inhibiting fat formation. When the P38 MAPK signaling pathway is activated, leptin production is inhibited, which may lead to fat accumulation and obesity.

The canonical Wingless/Integrated (Wnt) signaling pathway fundamentally governs the proliferation and differentiation of undifferentiated progenitor cells. Previous studies locally exposed mouse 3 T3–L1 fibroblasts to a Wnt signaling blocker, followed by a medium change to one devoid of supplements every other day. Furthermore, throughout each 2-day interval, 25 μmol of curcumin was consistently administered to the cells, accumulating to a total exposure duration of 2 days (75). *Ahn et al.*’s research revealed that curcumin suppresses the adipogenesis in 3 T3–L1 cells through stimulation of the conventional Wnt signaling pathway. Within the classic Wnt pathway, *β*-catenin interacts with TCF/LEF transcription factors, thereby profoundly impeding the transcriptional activities of PPARγ and C/EBP-*α*, both pivotal controllers of fat cell differentiation. Consequently, this mechanism ultimately curtails the maturation of 3 T3–L1 pre-adipocytes into fully developed adipocytes.

In addition, mTOR regulates cellular metabolism by integrating signals from nutrients, growth factors, and other environmental signals, and is essential for maintaining metabolic homeostasis and preventing disease. Obesity due to overnutrition may activate the mTOR pathway (76, 77), as evidenced by the increased activity of S6K and hyperphosphorylation of the translational repressor 4EBP in obese individuals (78).

#### 3.2.2 Transcription factor regulation

During adipocyte development, crucial transcription factors, such as C/EBP-*α*, C/EBP-*β*, and C/EBP-*γ*, along with peroxisome proliferator-activated receptors govern this differentiation stage (79, 80). PPARγ plays a pivotal biological role in directing adipocyte differentiation, steering this transformation through gene expression modulation. Activating PPARγ under specific circumstances accelerates adipocyte maturation, whereas the inability of PPARγ to function properly hampers efficient embryonic cell differentiation into adipocytes. Sterol regulatory element-binding proteins (SREBPs), such as SREBP-1, are crucial regulators of fat cell formation. They do this by managing the expression of essential enzymes for lipid synthesis, including acetyl-CoA carboxylase 1 and fatty acid synthase. It is well known that obesity produces proinflammatory cytokines that activate ERK and Cdk5. Inhibition of ERK or blockade of S273 accessibility by PPARγ ligands both prevent S273 phosphorylation and attenuate insulin resistance in obese wild-type mice and obese/ob mice (81, 82). PPARγ ligands, such as MRL24 and Mbx-102, are poor PPARγ agonists, but they effectively inhibit S273 phosphorylation and show strong antidiabetic activity *in vivo* (82).

*Woo et al.*’s study of 3 T3–L1 pre-adipocytes revealed that ellagic acid doses ranging from 5 to 50 μmol/L over 6 to 8 days reduced lipogenesis indicators—specifically, PPARγ and C/EBP-*α*—at both mRNA and protein expression levels. This regulation exhibited a dose-responsive pattern, consequently diminishing fat accumulation within cells in a dosage-dependent manner (83). Treatment of 3 T3–L1 cells with 0.1 to 10 μmol of ellagic acid or 30 μmol of curcumin over a span of 2 to 3 days led to a substantial decrease in the mRNA and protein expression of key adipogenesis players, namely PPARγ, C/EBP-*α*, SREBP-1c, and ACC (67, 84). PPARγ is induced during the differentiation of preadipocytes to adipocytes and is essential for this process. Without it, precursor cells cannot differentiate into mature adipocytes (85). PPARγ promotes adipogenesis in C/EBPα-deficient cells. Cells deficient in C/EBPα are able to differentiate into adipocytes.

**Figure 3:**
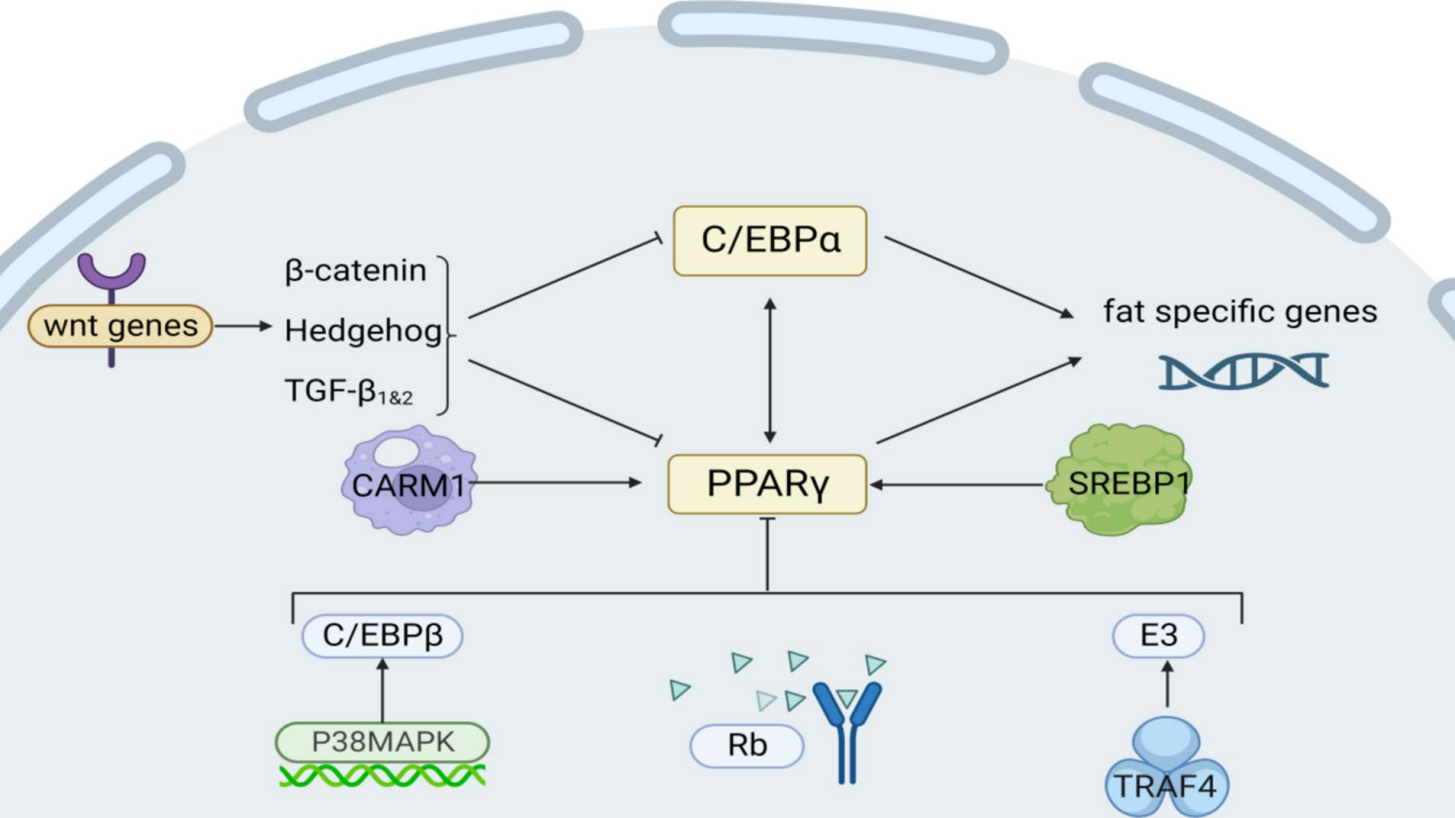
Intrinsic regulatory mechanisms affecting fat synthesis. Among them, Rb, P38/MAPK, and Wnt are signaling pathways; CARM1 and TRAF4 act on transcription factors PPARγ; and C/EBPα regulates lipogenesis genes.

Tumor necrosis factor receptor-associated protein 4 (TRAF4) acts as an E3 ubiquitin ligase to promote PPARγ protein degradation by ubiquitinating and activating the proteasomal degradation pathway (Figure 3), and YTHDF1 is able to specifically recognize and bind to the highly expressed form of TRAF4 mRNA, which can enhance the translation of TRAF4 and cause its intracellular protein level to increase. Curcumin inhibits the expression of AlkB homolog 5 (an m^6^A demethylase), reduces the demethylation of m^6^A-modified TRAF4 mRNA, and increases its intracellular level. *Chen et al.* discovered this by cloning the mouse TRAF4 gene (86). Moreover, the application of 10 μmol/L of ellagic acid to cells notably suppressed the expression of histone arginine methyltransferase 4 (CARM1), a vital histone arginine methyltransferase 4 for adipogenesis, particularly during cellular differentiation (87).

### 3.3 Fat metabolism regulation

Fat metabolism is a complex and sophisticated biochemical process, entailing several interrelated steps in the synthesis, storage, and degradation of a series of lipid substances, such as fatty acids, triglycerides, and cholesterol into energy. Several different biomolecules, such as enzymes and hormones, play an indispensable role in this process and collaborate to facilitate efficient fat metabolism and uphold the body’s energy balance (33).

#### 3.3.1 Catalysis of lipase

Lipase acts as a carboxylate ester hydrolase in the biochemical process, and its function manifests in its ability to catalyze the stepwise decomposition of triglycerides into glycerol and multiple fatty acid molecules (88). Adipocyte lipification’s typical pathway is catalyzed by three major lipases: hairy phosphatase domains containing-2/lipoacylglycerol lipase, hormone-sensitive lipase, and monoacylglycerol lipase. These three lipases work synergistically and degrade sequentially, eventually releasing the three fatty acids and glycerol. A mouse experiment conducted by *Kyeong Jo Kim et al.* revealed that ellagic acid impacted mice in a dosage-dependent fashion. Administration of 1, 2, and 4 mg/kg ellagic acid, respectively, led to a marked elevation in proteins linked to lipid metabolism, including triglyceride lipase—a pivotal fat metabolism enzyme—and phosphorylated hormone-sensitive lipase, alongside the significant protein PLIN1 (67).

#### 3.3.2 Regulation of AMP-activated protein kinase pathway

**Figure 4:**
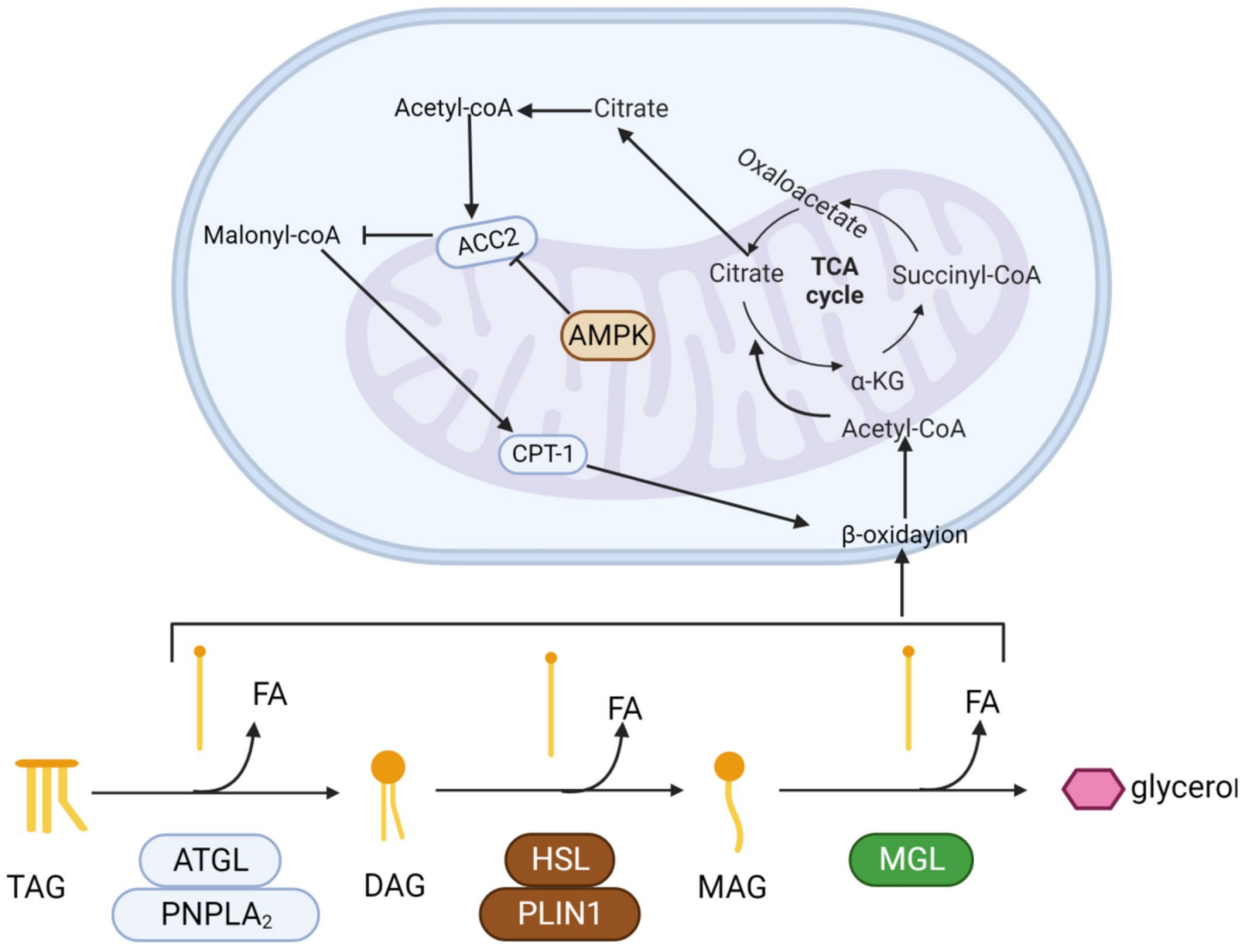
The process by which triglycerides are progressively hydrolyzed and oxidized. Triglycerides are hydrolyzed by esterase into glycerol and three fatty acids. Fatty acids enter the mitochondria and are progressively oxidized to acetyl-CoA by β-oxidase. Finally, acetyl-coA enters the tricarboxylic acid cycle. Activation of the AMPK pathway promotes β-oxidation.

Recent insights from research have revealed that AMPK precisely governs *in vivo* lipid synthesis, fatty acid catabolism, and oxidation by selectively phosphorylating critical substrates. It is heterologous inhibited to reduce positive oxidation of mitochondrial fatty acids. Blocking Acetyl-CoA carboxylase 2 (ACC2) lowers malonyl-CoA levels, enhancing CPT1 activity. This boost facilitates acyl-carnitine transport into mitochondria, stimulating fatty acid oxidation (Figure 4).

AMPK is a ubiquitously expressed serine-counting protein kinase that is essential for the maintenance of energy homeostasis (89). The AMPK signaling pathway can inhibit the ATP depletion pathway and has been linked to many different types of organic factors including muscle growth inhibitors and myosin (90, 91), who can participate in lipid metabolism through the AMPK signaling pathway. Resulting in the development of muscle-reducing obesity in the organism (92). Muscle growth inhibitors can inhibit protein synthesis by regulating the AMPK signaling pathway, leading to decreased muscle expression as well as browning of white adipose tissue (93). Myosin increases glucose uptake by activating the AMPK signaling pathway (94). The use of activated AMPK is currently approved for the treatment of diabetes (95).

When 3 T3–L1 pre-adipocytes were treated with 18 to 21 mg/g of ACC2, an upsurge was observed in AMPK and its activated form, p-AMPK. Consequently, the expression of CPT1, an enzyme central to mitochondrial fatty acid oxidation, rose notably. This enhancement expedited fat and triglyceride breakdown, thereby stimulating fatty acid oxidation and augmenting energy expenditure.

### 3.4 Alleviating insulin resistance

Insulin binds to receptors on liver, skeletal muscle, and fat cells’ membranes, effectively enhancing glucose absorption and usage by these tissues. Insulin resistance causes cells to become less sensitive to insulin as well as instigating changes in glucose metabolism (e.g., uptake or storage).

#### 3.4.1 Regulation of PI3K/AKT signal pathway

PI3K is a family of lipid kinases that phosphorylate phosphatidylinositol, a component of eukaryotic cell membranes (96). There are three classes of PI3K: classes I, II, and III. AKT consists of three structural domains: pleckstrin homology (PH), middle kinase, and regulatory carboxy-terminal domain. of these, AKT2 is predominantly expressed in skeletal muscle, adipose tissue, and liver insulin-sensitive tissues such as skeletal muscle, adipose tissue and liver (97). Polyphenols may alleviate obesity by targeting PI3K/AkT. Quercetin down-regulates pro-inflammatory cytokine expression by altering the PI3K/Akt signaling pathway (98), and ellagic acid protects HUVEC from PI3K/Akt activation-mediated apoptosis (99).

Transcriptional activator 3 (STAT3) signaling activation stimulates a negative feedback response by inducing suppressors of cytokine signaling factor to inhibit the insulin /IGF-1 receptor JAK1/2. PI3K/AKT signaling downstream of the insulin/IGF-1 receptor is reduced by Forkhead box O—specifically, Forkhead box O1 (FOXO1)—is the primary target of AKT and affects energy balance throughout the body. FOXO1 and peroxy proliferation-activating receptor-Coactivator 1a work together to regulate gene expression and increase glucose production and fatty acid oxidation. In addition, FOXO1 also up-regulates phosphoenolpyruvate carboxykinase and glucose-6 phosphatase, two enzymes that play key roles in the glucose synthesis pathway, thereby promoting increased glucose production. Thus, AKT directly inhibits FOXO1 and reduces glucose levels.

In a curcumin therapy experiment, *Ren Li et al.* found that curcumin inhibited the activation of signal transduction and STAT3 in human adipocytes after injection of 10 mg/kg curcumin in mice. The STAT3 signaling pathway is pivotal in the physiological and pathological regulation of lipid and carbohydrate metabolism and immune responses, notably regarding issues of insulin resistance in obesity. This signaling network is considered to be a key pathway mediating inflammatory response and is closely related to the regulation of immune function (100).

Research has demonstrated that oral administration of 25 to 50 mg/kg ferulic acid can notably lower blood sugar levels and help alleviate insulin resistance. Ferulic acid’s improvement of blood glucose and insulin resistance may be related to its effect on FOXO1 protein. Research indicates that FOXO1 can bind directly to the glycogen synthase gene’s promoter, thereby activating glucose production. This pathway represents a possible therapeutic target for ferulic acid’s beneficial actions. In conclusion, FA significantly reduced elevated blood glucose and serum leptin levels, decreased insulin resistance, and increased serum lipocalin levels (101). Interestingly, however, when Chowdhury S gave rats four different doses of ferulic acid (10, 30, 50, and 70 mg/kg body weight) every day for 8 weeks, it was found to work directly by activating the PI3K/AKT/Glucose Transporter 4 (GLUT4) signaling pathway (102).

#### 3.4.2 Regulation of the AMPK pathway

**Figure 5:**
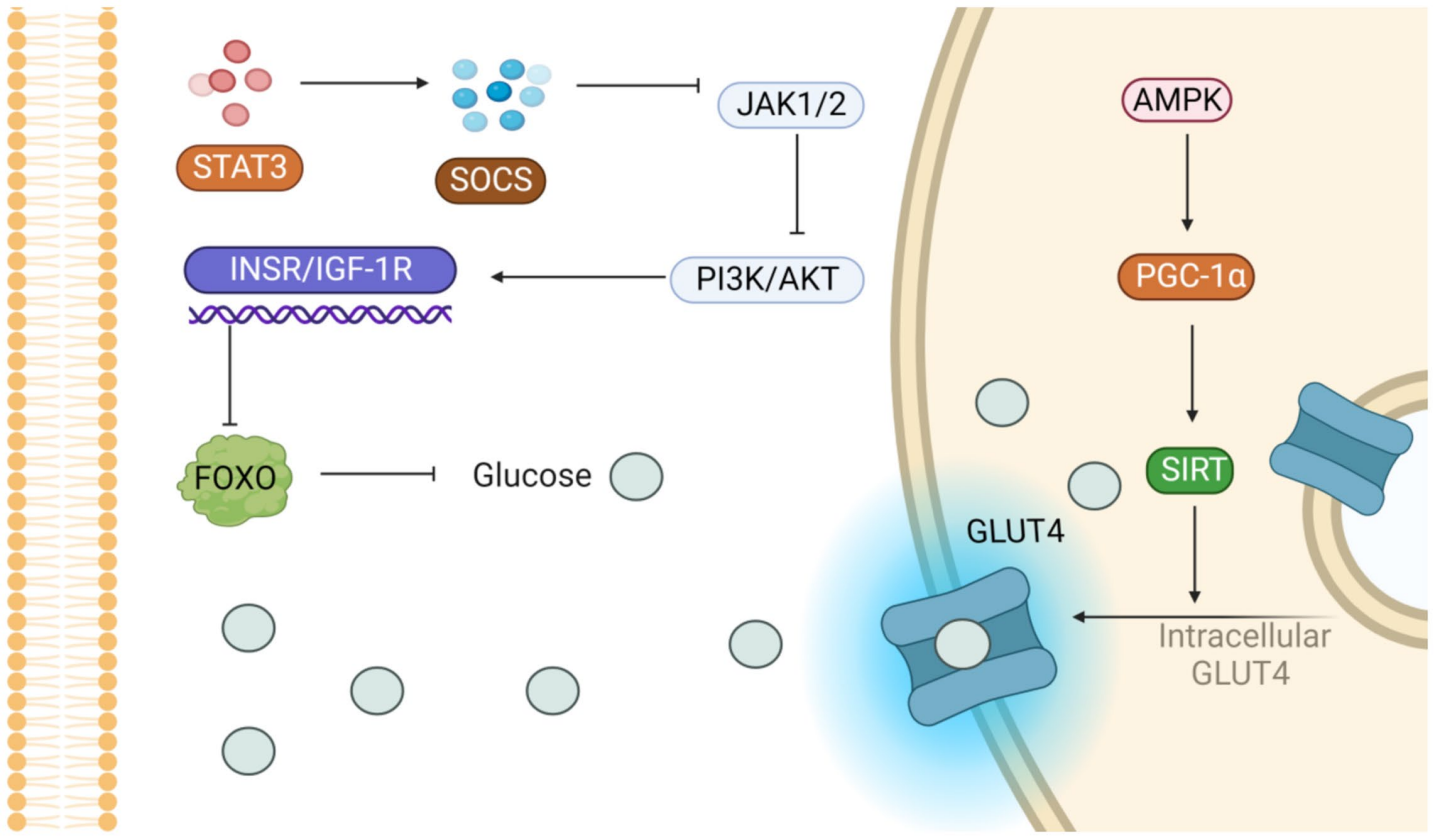
Potential signaling pathways that alleviate insulin resistance. On the one hand, the activation of PI3K/AKT pathway inhibits glucose production; on the other hand, the activation of AMPK pathway accelerates GLUT-4 translocation and promotes glucose entry into cells.

Activation of the AMPK pathway increases the expression of GLUT4 and promotes its translocation, while the increase of CPT1 and PGC1α promotes mitochondrial *β* oxidation and inhibits lipogenesis and triglyceride (TG) resynthesis to regulate energy homeostasis (Figure 5) (103). *Jing Dong et al.* acquired a cohort of 4-week-old male C57BL/6 mice, acclimatized them for a week, and then allocated them randomly into three groups (each with 8 mice). These groups received either a low-fat diet (LFD) or an HFD, with the HFD group further receiving 0.1% quercetin (of at least 98% purity) supplementation. Quercetin activates AMPK and its subsequent extracellular signal-regulated kinase pathway, which heightens glucose transporter 4’s movement into skeletal muscle and fat tissues. This fosters fat cells’ glucose uptake, enhancing insulin sensitivity and glucose metabolism (104).

*Okla et al.* discovered that exposing mature human fat cells or liver cells to 10 μmol/L (equivalent to 3 μmol/mL) of ellagic acid led to a considerable decline in lipid accumulation within these cells after culturing them for 3 days. Using radiolabeled substrates, ellagic acid was shown to significantly inhibit intracellular TG regeneration and esterification and to enhance the oxidative metabolism of ferulic acid. These effects are intimately tied to AMPK’s function, as AMPK activation is pivotal in energy homeostasis regulation. It enhances cellular energy efficiency and sustains the brain–blood barrier’s energy balance by curtailing fat synthesis, decreasing triglyceride formation, and fostering the ferulate oxidation pathway (105).

### 3.5 Alleviating inflammation

Adipose tissue experiences both hyperplasia (an increase in cell numbers) and hypertrophy (enlargement of cells), which can impact the tissue’s capacity to expand and receive oxygen, potentially resulting in hypoxia and triggering the activation of hypoxia-inducible factors (35).

#### 3.5.1 Regulation of AMPK pathway

When the AMPK signaling pathway is activated, it further inhibits NF-κB, which, in turn, promotes the main transcription factor Heme Oxygenase-1(HO-1) in the pro-inflammatory response, which, in turn, suppresses inflammation. *Ahad et al.* have shown that ellagic acid can suppresses NF-κB, a key transcription factor implicated in inflammation, to prevent the happening of inflammation. This action improves abnormal lipid levels in test rats and exerts favorable therapeutic outcomes for diabetic kidney disease. Administering escalating doses of electroacupuncture (ranging from 20 to 100 mg/kg body weight over 14 days) to 3 T3–L1 fat cells notably decreased the expression of the NF-κB-p65 subunit, transforming growth factor *β*, and fibronectin. Concurrently, elevated HO-1 enzymatic activity was detected (106).

#### 3.5.2 Inhibition of inflammatory factors

**Figure 6:**
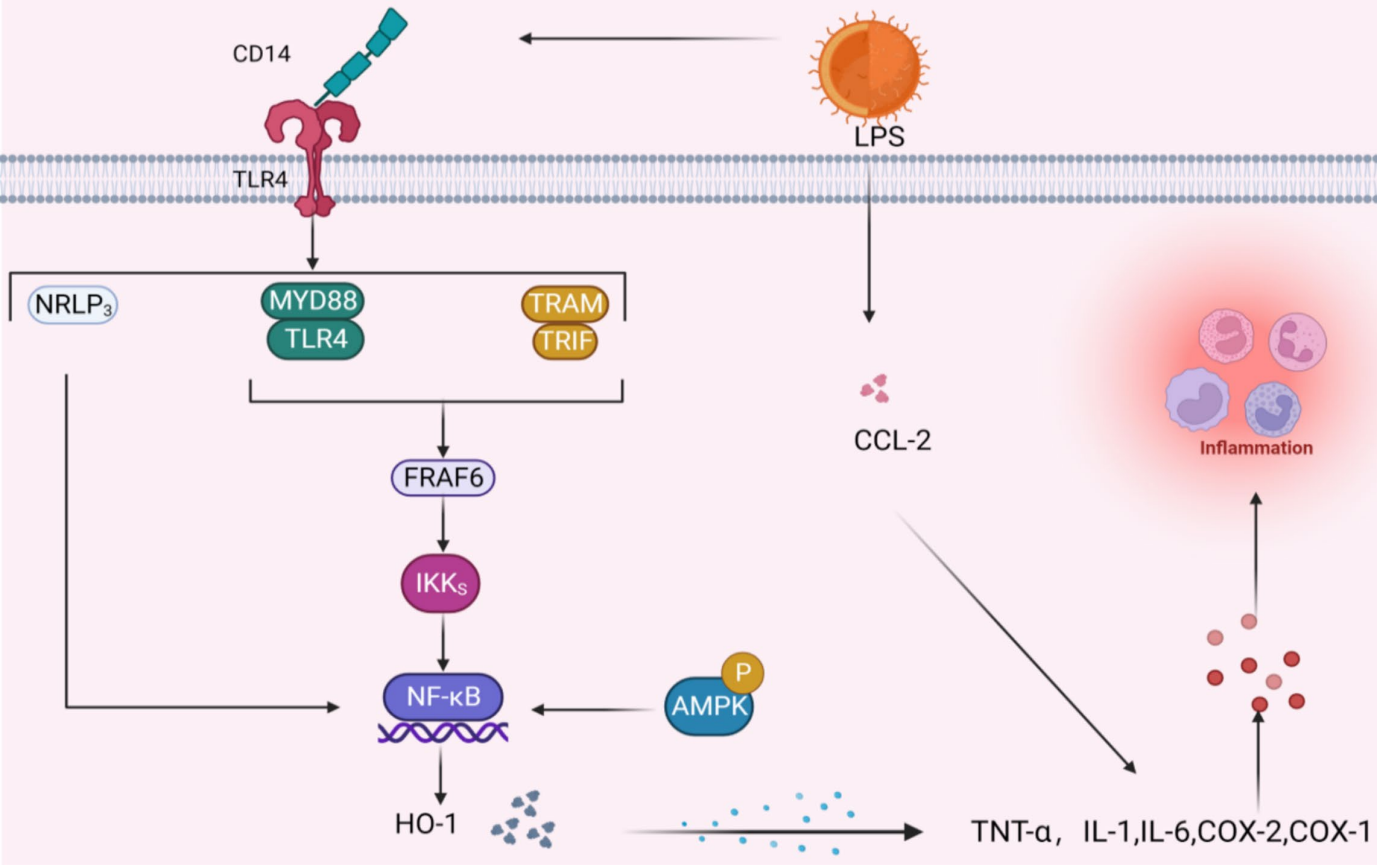
Potential signal mechanism of inhibiting inflammatory factors. When LPS binds to TLR4 via CD14 three pathways are activated, the common result is to activate the NF-κB pathway; the activation of AMPK signal will also produce the same effect. The NF-κB pathway further acts with HO-1 to promote the release of inflammatory factors, and LPS can also directly act on inflammatory factors through CCL-2.

As adipose tissue expands, both enlarged adipocytes and resident immune cells undergo a transformation in their behavior: they shift from releasing primarily anti-inflammatory and protective substances to generating substantial amounts of inflammatory adipokines and other cytokines that promote inflammation, impacting both the local area and the entire body (Figure 6).

Quercetin, catechin, and similar polyphenolic compounds stimulate the body’s production of interleukin-10, an anti-inflammatory agent. This action effectively curbs the activities of pro-inflammatory molecules, such as tumor necrosis factor-*α* (TNF-α) and interleukin-1, thereby fine-tuning the body’s inflammatory response equilibrium at a molecular level (107, 108). Past research has indicated that ellagic acid efficiently modulates genes tied to inflammation. In lab settings, it directly suppresses macrophage and fat cell inflammation by lowering the lipopolysaccharide (LPS)-induced transcription of pro-inflammatory substances TNF-*α*, IL-6, and CCL2 (103). Studies have demonstrated that curcumin, taken orally in 300 mg doses twice daily, can directly bind and inhibit the activity of inflammatory cytokines, including Cyclooxygenase-1, Cyclooxygenase-2, and MMP. *Salazar-Lopez et al.* discovered that 0.5% ferulic acid decreased daily weight increase, fat accumulation, and WAT weight in obese C57BL/6 J mice in addition to hindering liver fat buildup and reducing the release of inflammatory markers IL-6 and TNF-α (109).

### 3.6 Regulating gut microbiome

Obesity onset is accompanied by marked shifts in the gut microbiome’s composition, ratio, and variety. Research reveals that ingesting ellagic acid or foods abundant in ellagic acid can mitigate obesity and its associated metabolic issues. This is achieved by altering the intestinal flora’s metabolism, which subsequently influences the production of microflora-derived urea. In a human study, healthy participants drank 1 liter of pomegranate juice containing 4.37 grams of ellagic acid per liter daily over 5 days. The results indicated that ellagic acid reduced obesity and metabolic disorders by boosting microbiota diversity and reducing Firmicutes, a bacteria group responsible for producing urolithin (a metabolite derived from ellagic acid in the gut). This modification in gut bacterial profile and function positively altered the gut microbiota’s metabolism (103).

In models of obesity caused by HFDs, the balance of GM communities is disrupted, and the functional integrity of the gut barrier is compromised. Therefore, in the obese state, the bacterial endotoxin LPS in the circulatory system can cross the intestinal barrier that is damaged by obesity and spread through the blood circulation to all tissues of the body. In the WAT of obese individuals, in particular, LPS can activate a series of anti-inflammatory signaling pathways (38). *Islam et al.* recently explored how the combination of a HFD with curcumin might interact in terms of management of inflammation linked to obesity in fat tissue. They also examined alterations in gut microbe populations during this intervention. Over a 14-week period, male B6 mice were divided into two dietary groups: those fed a high-fat diet comprising 45% of calories from fat (HFD), and others given a high-fat diet supplemented with 0.4% curcumin, labeled as HFC. The findings revealed that mice on the curcumin-supplemented HFD had notably diminished fat buildup in WAT and reduced overall macrophage infiltration compared to those consuming the HFD without curcumin (110). Curcumin appears to alleviate HFD-induced obesity-related inflammation in fat tissues by adjusting the gut microbiota composition and stimulating the curcumin-O-glucuronic acid metabolism pathway.

Studies have revealed that obesity is associated with changes in the relative abundance of the two major intestinal bacterial clades, *Bacteroidetes* and *Firmicutes*, and increased levels of *actinobacteria*. Rats were allocated randomly to four groups, with some fed a HFD at a daily dosage of 30 mg per kg of body weight and others receiving additional quercetin. The results showed that quercetin supplementation significantly affected GM community structure at different hierarchical levels, as evidenced by the reduction of Firmicutes and Bacteroidetes, as well as the inhibition of the proliferation of bacteria such as Erysipelaceae, Bacillus, and Baculobacterium, which are commonly associated with HFD-induced obesity. In essence, quercetin is effective in mitigating the gut microbiota dysbiosis triggered by an HFD (111).

*Field et al.*’s study involved feeding C57BL/6 J mice either an LFD or an HFD, with some high-fat groups also receiving ferulic acid via gavage at a dosage of 100 mg/kg for a duration of 12 weeks. Ferulic acid was shown to reduce weight gain and correct the HFD-induced gut microbiota imbalance, and significantly promoted the accumulation and receptor expression of short-chain fatty acid-producing bacteria in the intestine, including *Olsenella, Eisenbergiella, Dubosiella, Clostridiales_unclassified*, and *Faecalibaculum*, reduced endotoxigenic or obesity-associated bacteria, decreased LPS, and inhibited the colonic TLR4/NF-κB pathway. Therefore, this shows that ferulic acid can mitigate colon barrier dysfunction and intestinal inflammation by regulating GM (112). Hereditarily leptin-deficient obese (OB/OB) mice were administered 0.5% ferulic acid over a 9-week period. In the control experiment, FA exhibited superior weight loss to that of wild-type (WT) control mice. Following treatment, researchers noted that ferulate did not alter the total levels of obesity-linked or anti-obesity genes in the OB/OB mouse model. It also had no considerable impact on the diversity of GM communities in these obese mice (113), suggesting that ferulic acid does not influence obesity resulting from genetic leptin deficiency.

Relevant clinical trials could further demonstrate the effects of polyphenols on the gut microbiota. Water-soluble tomato extract, a polyphenol-rich natural product, was used in a randomized, double-blind, placebo-controlled trial of overweight and obese adults in which 22 subjects received 2 × 150 mg of Fruitflow or placebo (maltodextrin) daily for 4 weeks, with 6 weeks between interventions. The results found that Fruitflow reduced fasting blood glucose and altered intestinal flora. The results suggest that polyphenol-rich extracts can reduce plasma TMAO in overweight people, which is associated with gut microbial regulation (114).

In addition, it is worth mentioning that recent studies have shown that various polyphenols have different effects on microbial composition and function (115), and that quercetin may be able to increase *Lactobacillus* (116), a genetically modified organism found to be biologically relevant in the biotransformation and degradation of curcumin (117).

In addition to their antioxidant, antitumor, and anti-inflammatory properties, polyphenols have the ability to prevent or delay the formation of senescence and slow down the aging of skin appearance and function (118). Several *in vitro* studies have shown that polyphenol treatments slow cellular senescence and that an increase in SASP is one of the hallmarks associated with aging. In one study, polyphenols such as quercetin and kaempferol all inhibited the secretion of the SASP markers IL-6, IL-8, and IL-1β, to slow down cellular aging (119).

## 4 Limitations of polyphenols in regulating obesity

Numerous studies have demonstrated that quercetin, ferulic acid, curcumin, and ellagic acid as well as other polyphenols have obvious limitations, including instability, low solubility, fast metabolism, poor absorption, and poor bioavailability and require further improvement.

### 4.1 Curcumin

Turmeric is a versatile spice that has garnered considerable attention across medicine, science, and cuisine. This herbaceous, rhizomatous perennial, *Curcuma longa*, belongs to the ginger family and holds particular importance as a primary source of curcumin (120). In recent times, curcumin has piqued the interest of scientists and the general public alike, owing to its prospective health benefits, which include anti-inflammatory, anti-diabetic, anti-carcinogenic, and anti-aging properties. It has exhibited significant application potential for the promotion of wound healing, combating arthritis and treating Alzheimer’s disease (121–124). Nonetheless, curcumin’s bioavailability is limited due to its instability in water, poor solubility, rapid metabolic breakdown, and low absorption rate (125), which greatly restricts its practical application as an effective therapeutic drug, and it has not yet been approved as a formally marketed therapeutic drug. To overcome this limitation, delivery of polyphenols by nanocarriers is a viable strategy, such as Phytosome, Lipid-Based Nanoparticles, Niosomes, Protein-Based Nanoformulations, Polymeric Nanoparticles, and Micelles (126). Perhaps in the future, through these techniques and tools, it will be possible to see the application of Turmeric for the treatment of obesity in the clinic.

Curcumin’s stability under physiological conditions is poor; for example, it has been reported that its half-life is less than 10 min at 37°C and neutral pH (7.2) (127). Curcumin is degraded via two pathways: solvent-decomposition and photodegradation (124, 128). During solvent-initiated dissolution, solvent molecules sometimes undergo nucleophilic substitution or elimination reactions. In particular, the *α* – and *β*-unsaturated ketones present in curcumin’s structure undergo nucleophile attack, a process specifically referred to as the Michael addition reaction.

In the solvation process under alkaline conditions, curcumin’s heptanone moiety undergoes significant degradation with a degradation rate of up to 90%. In this process, a series of decomposition products, including vanillin (129). Meanwhile, curcumin is autoxidized through a free radical chain reaction, in which oxygen is incorporated and converted to cyclopentanedione. In addition, several studies have demonstrated that curcumin is sensitive to light, irrespective of its state (i.e., solid or solution). Owing to its photosensitive properties, curcumin must be stored in darkness to prevent it from degrading as a result of light exposure (127).

Curcumin’s low water solubility and unstable nature lead to minimal dissolution in the digestive system, thereby restricting its effective biological action. It should be noted that curcumin has a significant hydrophobic property, and its theoretical maximum solubility is roughly 11 ng/mL. To break through this barrier, researchers have adopted a series of advanced preparation technologies, such as microcapsule encapsulation, micelle system construction, and recombination with different carrier molecules (130, 131). To enhance curcumin’s water solubility and stability in the body, researchers are attempting to boost its bioavailability.

After it enters the blood circulatory system, curcumin undergoes a rapid metabolic process and is converted to a biologically inactive form. In this metabolic process, curcumin is gradually reduced to a series of derivatives, including dihydrocurcumin, tetrahydrocurcumin, hexahydrocurcumin, and octahydrocurcumin. Subsequently, the above forms, along with curcumin, are converted to conjugated glucose and sulfate in the second phase, forming a non-biologically active glucose and sulfate conjugation (132).

Another considerable challenge to curcumin’s therapeutic potential is its poor bioavailability, of which poor absorption and fast metabolism are the main causes. Although curcumin is a compound that appears to be tolerated well when taken in a single, large oral dose, in a study by *Law et al.*, the administration of a single oral dose of up to 12,000 mg of curcumin showed no evident adverse effects (133). Moreover, at high doses, oral curcumin and turmeric are not reproductively toxic to animals. Human research confirms curcumin’s safety, with no toxicity observed at a daily oral dose of 6 grams taken over a period of 4 to 7 weeks. In addition, oral formulations of 500 mg bioavailable curcumin twice daily for 30 days are safe in humans but are still rarely tested. Thus, further research is necessary to establish a safe and efficacious dosage. Clinical studies have reported that curcumin is not detectable in serum samples from the vast majority of participants, including those who consume a dose of 12 g per day, which is not surprising because curcumin itself is not efficiently absorbed by the body. Curcumin’s low solubility and instability in the gut limits its effectiveness, spurring researchers to explore enhancements such as microencapsulation, micelle technologies, and binding to supportive molecules. These strategies are aimed at boosting curcumin’s water solubility, stability, and, ultimately, its bioavailability. The scientific community is actively seeking solutions to address curcumin’s low absorption issue (131).

In rats, only 60% of the dose of oral curcumin is adsorbed, and minimal amounts (<20/g/tissue) are detected in the liver and kidneys 15 min to 24 h after administration (134). The results of another test showed oral curcumin given in doses of 12 g/day, but in this case, the final serum curcumin level was only 50 ng/mL. Extensive research indicates that much consumed curcumin is expelled from the body as metabolites, a phenomenon that is likely to be closely related to the P-glycoprotein transport system and the first-pass effect mechanism in the liver. Curcumin is a substrate for P-glycoprotein, a transmembrane drug-dependent secretory pump that eases curcumin from the intestinal membrane, thereby limiting its permeability. In addition, the first pass effect of liver result in some metabolism of curcumin in the intestinal mucosa and liver, lead to reduce drug absorption (132), Only a small amount of curcumin enters the blood supply, which is much lower than the concentration required to inhibit most of the targets of curcumin, so the therapeutic effect is much lower than expected (135). In light of the above challenges in biological transport, researchers are not only developing strategies, such as microencapsulation, micellar technology, and binding carrier molecules, aimed at improving curcumin’s aqueous solubility and *in vivo* stability but are also exploring how to overcome the P-glycoprotein-mediated drug efflux and other physiological barriers to effectively improve curcumin’s actual biological utilization level of curcumin. At present, researchers are conducting in-depth research to identify new methods and strategies to solve this scientific problem (70).

Fortunately, various approaches to improving curcumin’s bioavailability have already been investigated. Such methods include concomitant use of piperine to interfere with glucose binding and the encapsulation of curcumin in liposomes. Another key approach involves the miniaturized nano-delivery systems developed for curcumin. Another method is the combination of curcumin with phospholipid molecules through chemical synthesis. The fifth strategy is the design and synthesis of compounds with similar structural characteristics to those of curcumin. Such compounds have the reported advantages of rapid uptake *in vivo* and short plasma peak concentration half-life, thus effectively improving the actual utilization rate of curcumin in the human body. The research team tirelessly pursues and applies a range of strategies, including these novel techniques, to surmount curcumin’s inherent low bioavailability challenge, with the goal of enhancing its absorption and biological effectiveness in humans (129), further effects remain to be verified but may nevertheless serve as a major breakthrough.

### 4.2 Quercetin

Quercetin is derived from a wide range of sources, particularly foods (120). This includes various plant-based products, such as vegetables, fruits, berries, nuts, and beverages (136). Quercetin is found in varying levels in honey sourced from different plants, with the highest concentration found in raw edible part of capers and the lowest concentration in black or green tea (Camellia) edibles (137). Quercetin exhibits anti-inflammatory, anti-proliferative, and anti-angiogenic properties, but its low water solubility poses challenges (138). Its clinical use in cancer therapy is restricted due to issues such as poor absorbability, instability, brief duration of effect, and limited bioavailability. Quercetin has been shown to be unstable and poorly absorbed in the GI tract. Only a small amount of quercetin can be effectively taken up by the GI mucosa after ingestion. When quercetin enters the bloodstream, it is metabolized in the liver by means of a two-stage process (139). This series of metabolic reactions will eventually produce metabolites that can circulate in the blood and further diffuse into various tissue structures in the human body. However, the liver has a fast metabolism, which makes the biological half-life of quercetin as short as 1–2 h. *Mullen et al.* conducted a study analyzing and measuring key quercetin metabolites in plasma and urine following onion consumption. Following ingestion of quercetin, its metabolites are able to rapidly enter the circulation and be detected within 30 min; however, they are excreted from the body in large quantities over the next 24 h (140). This phenomenon vividly illustrates the dynamic process by which quercetin is rapidly cleared from the blood and implies that quercetin has a short plasma half-life (139). However, some individuals exhibit high levels of quercetin metabolites, with a notably slow elimination process, reporting a half-life of 11 to 28 h (141). The average final half-life of ceratocertin is approximately 3.5 h (142). The total recovery rate of quercetin in urine, feces, and exhaled breath can vary significantly between individuals (143, 144).

This variability is attributed to factors such as low absorption, extensive metabolism, and/or swift elimination rates (145). Quercetin is rapidly metabolized in the human body, resulting in its low bioavailability; *Conquer et al.* initiated a clinical trial, the first of its kind, that compared the impact of various quercetin forms and doses—1 gram of free quercetin against 200 milligrams of rutin capsules—on plasma quercetin levels. They also examined the potential effects of these formulations on cardiovascular disease risk factors. Following 28 days of quercetin supplementation, the plasma quercetin concentration saw a notable rise from 0.1 μmol/L to 1.5 μmol/L (143). However, in the conducted comparative studies, no statistically significant distinctions were observed between the groups in terms of cardiovascular function metrics and thrombosis-related risk factors, such as platelet aggregation capacity, platelet-mediated thrombophilia, blood pressure level, and resting heart rate. In this study, all participants had normal health status and blood pressure levels, a fact that may help explain why no substantial treatment effect was evident. In another relevant study, *Edwards et al.* demonstrated that consuming 730 mg of sugar-free quercetin effectively reduced blood pressure in individuals with stage 1 hypertension (146). However, in prehypertensive individuals, the same dose did not show the same significant antihypertensive effect. Moreover, a separate study offered further insight and complementary data on the complex relationship between quercetin dosage and cardiovascular health (139). In a medical study, a total of 15 participants were instructed to continue taking their original antihypertensive medications (147). The study focused on assessing the impact of these medications on oxidative stress and inflammation markers in humans, particularly—though not exclusively—emphasizing tumor necrosis factor-alpha (TNF-*α*) and C-reactive protein. The results demonstrated that these markers did not change significantly over the course of the trial (148). Interestingly, however, individual studies demonstrated that quercetin also had a significant therapeutic effect. In one study, 500 mg of quercetin supplementation in women with type 2 diabetes led to a reduction in inflammatory markers TNF-α and IL-6, both of which are strongly associated with cardiovascular disease, indicating its potential benefits (149). Although the current data show a clear inhibitory effect, in-depth experimental validation is needed to reveal the presence or absence of the opposite effect.

Thoroughly understanding the molecular processes controlling drug delivery is crucial for advancing the creation of novel and effective therapeutic approaches. To overcome the limitation of low *in vivo* utilization of quercetin, the delivery of quercetin in the form of nanoconjugates has been proposed as a strategy. Nanoconjugated quercetin has garnered considerable interest owing to its distinctive characteristics, which enhance quercetin’s absorption and distribution in the human body, thereby improving its efficacy (150), and may enhance its intervention efficacy on cardiovascular disease-related risk factors (151). Thus, it is expected that the optimal matching between quercetin’s dosage and efficacy can be achieved. This advancement may broaden quercetin’s application for preventing and treating cardiovascular diseases (152). In fact, nanotechnology has been demonstrated to deliver quercetin with superior efficacy in populations with different blood pressure stages, which has enhanced our understanding of the diversity of quercetin dose–response relationships (153). The efficacy and level of quercetin optimization via nanoconjugation largely hinges on the choice of carrier, including liposomes, silver-based nanomaterials, silica nanoparticles, poly-D-L-lactic acid copolymers, or PLLA nanoparticles (139, 140, 145). Polymer micelles and chitosan nanoparticles are among the carriers that significantly influence quercetin’s *in vivo* delivery efficiency, stability, and targeting. These carriers are crucial for determining the ultimate therapeutic outcomes. Each vector has its own characteristics, which may uniquely enhance quercetin’s bioavailability or promote the accumulation of quercetin in the key tissues and cells involved in related cardiovascular risk factors to maximize its therapeutic effect against various cardiovascular risk factors.

Therefore, in the process of developing quercetin nanoconjugates, the selection of appropriate drug carriers is a core research task that will help researchers to determine the most effective quercetin delivery scheme, thus enhancing its accuracy and effectiveness in preventing and treating cardiovascular diseases (154).

### 4.3 Ellagic acid

Ellagic acid, a polyphenol, is derived from various sources. Research indicates that ellagic acid has been isolated from families that include *Rosaceae, Saxifragaceae, Ericaceae, Combretaceae, Anacardiaceae, Vitaceae, Punicaceae, Juglandaceae, Fabaceae, Euphorbiaceae, Sapindaceae,* and *Simarubaceae* (155). Ellagic acid is particularly abundant in berries from the Rosaceae family, like raspberries, cherries, and strawberries (155). Recent studies have uncovered the numerous pharmacological benefits of ellagic acid, including its antioxidant properties, anti-inflammatory effects, lipid metabolism regulation, virus activity inhibition, anti-angiogenesis, and potential anti-cancer effects (156, 157). Ellagic acid has various bioactive mechanisms, including free radical elimination for antioxidant protection, regulation of lipid metabolism, and interference with the activities of proteins that cause fibrotic reactions. In addition, it inhibits the dysfunction of hepatic stellate cells and myofibroblasts, impedes viral replication in the liver, and facilitates the expression of factors with growth inhibitory effects while participating in the precise regulation of transcription factor activity (158). The oral and blood supply of these components is very low, diminishing ellagic acid’s efficacy (159).

Although it is a small molecule, ellagic acid has poor water solubility. It can exist in free form, as ellagic glycosides, or in bound forms. Under normal physiological conditions, ETs are hydrolyzed to produce ellagic acid, which is not directly absorbed in the stomach and small intestine (160, 161). Ellagic acid that is unchanged proceeds to the large intestine, where intestinal microflora metabolizes it into urolithin, which is subsequently absorbed into the bloodstream. The low bioavailability of ETs may be due to their large size and high polarity as well as the presence of C-C bonds. ETs undergo facile hydrolysis in the stomach and duodenum, liberating ellagic acid with diminished bioavailability. This is attributed to its low aqueous solubility, extensive pre-absorption degradation, permanent DNA adherence, and formation of insoluble complexes with calcium and magnesium ions (162, 163). This significantly hampers absorption between cells and can impact their transportation (164).

Ellagic acid also had extremely poor bioavailability, with 10% of the total ellagic acid dose detected in urine and feces when administered to mice (from raspberries or pomegranates) outside of the injection. However, almost no traces of it in the blood or tissue (165). In a recent human study, scientists investigated the immediate consumption of 800 mg of pomegranate extract by healthy volunteers to assess its absorption in the body. The results showed that the highest plasma concentration (Cmax) of ellagic acid reached a mean value of 33.8 ± 12.7 ng/mL (166) at the time point of 1 h after ingestion, and the corresponding time to peak (tmax) was recorded (167). In a pharmacokinetics study, peak blood levels of ellagic acid (Cmax) occurred one-hour post-consumption of 180 mL pomegranate juice with 25 mg ellagic acid and 318 mg related compounds. The peak concentration (Cmax) of ellagic acid in circulation reached 32 ng/mL, or 0.1 μM, and it was cleared from the bloodstream over the period 4 h (168). In another study in Spain, six healthy volunteers were provided one liter of pomegranate juice, despite much higher doses, ellagic acid was not observed in plasma for 4 h after juice intake (168–170).

In recent years, a series of improvements have been made to overcome the limitations of ellagic acid, covering different strategies such as chemical structure modification, encapsulation of ellagic acid in nanoparticles as delivery carriers, and molecular level dispersion in polymer media (171).

### 4.4 Ferulic acid

Ferulic acid, a ubiquitous plant phenolic, is particularly prevalent in plants like Umbelliferae, Ranunculaceae, and Grasses, including species such as Angelica, Ligusticum chuanxiong, Cimicifuga, Sparganium rhizome, and Reed root. Frequently bound to lignin and polysaccharides as part of plant cell walls, it is seldom found in a free form (172). Ferulic acid is a crucial active component found in various traditional Chinese medicines. It has demonstrated diverse biological activities, particularly in combating oxidative stress, inhibiting inflammatory responses, safeguarding vascular endothelium against injury, preventing fibrosis progression, regulating cell apoptosis, and inhibiting excessive platelet aggregation (173, 174). Owing to these characteristics, ferulic acid is widely used and studied in the cosmetic industry (172). However, its therapeutic potential is limited by its low solubility and bioavailability.

After ferulic acid is absorbed by the stomach (175), it rapidly forms complexes in the liver with glucose, sulfuric acid, and gluconate sulfate (176). After a single administration, ferulic acid shows a wide distribution property in various parts of the body. Approximately 4% of the total dose is found in the gastric mucosa, 10% in plasma, liver, and kidneys, with approximately 53% distributed across other bodily tissues. However, ferulic acid’s bioavailability is relatively low as it quickly binds in the liver upon entering the bloodstream (176).

In-depth pharmacokinetic analysis indicates that ferulic acid shows rapid absorption and excretion after a single oral intake. However, its bioavailability is not ideal, which undoubtedly poses an urgent problem for future research. It is worth noting that well-designed synergism strategies can effectively enhance ferulic acid’s utilization efficiency *in vivo* and improve its therapeutic effect. Therefore, the core of future research will focus on finding drugs that synergistically enhance ferulic acid bioavailability. Meanwhile, it must be noted that a drug’s bioavailability is typically closely related to the chemical structure of the drug itself. This also means that by adjusting and optimizing ferulic acid’s chemical structure design, its bioavailability *in vivo* can be further effectively enhanced (102) (Table 1).

**Table 1:** A brief summary of experiments on four polyphenols.

Number	Author	Design	Result	Reference
1	Christopher D. Lao, Mack T. Ruffin 4th, et al.	Human model Healthy volunteers were given from 500 to 12,000 mg of incremental doses. Safety was assessed within 72 h	Only minor traces of curcumin were identified in two individuals who took 10,000 or 12,000 mg, while it remained undetected in others. A singular, large oral dose of curcumin seems to be largely well-tolerated.	(133)
2	William Mullen, Christine A. Edwards, Alan Crozier, et al.	Human model healthy volunteers consumed onions lightly cooked with 275 micromoles of flavonols, chiefly quercetin-4′-glucoside and quercetin-3,4′-diglucoside. Plasma and urine samples were gathered over 24 h	Plasma showed quercetin metabolites within 30 min of intake, yet a substantial portion was expelled within 24 h, suggesting quick elimination and a brief blood half-life for quercetin.	(139)
3	Xiao Chen, Ophelia Q., P. Yin, et al.	Rat model oral contains quercetin and its metabolites of bile, evaluated the enterohepatic circulation again	Quercetin’s bioavailability reached a mere 5.3%, yet its overall absorption efficiency was 59.1%. Post-ingestion, roughly 93.3% of quercetin underwent gut metabolism, with just 3.1% processed in the liver.	(177)
4	I Ueno, N Nakano, I Hirono et al.	Male rats model (ACI) The distribution, metabolism, and excretion of quercetin were studied by autoradiography and/or radioactive quantification and chemical analysis	Upon oral intake of [14C]-quercetin, approximately 20% is absorbed by the gut, over 30% is metabolized into 14CO2, and around 30% is eliminated unchanged in feces. Following absorption, [14C] quercetin, mostly as glucuronide and sulfate conjugates, is swiftly excreted via bile and urine within 48 h.	(178)
5	T Walle, U. K. Walle, et al.	Human model Healthy volunteers were given 1.85 MBq (50 microCi) of (14)C-quercetin, orally (100 mg, 330 micromol) and intravenously (0.3 mg, 1 micromol). Sequential plasma, urine, and stool samples were collected over 72 h.	the total recovery of the (14)C-quercetin doses, in particular after oral administration, was very low.	(179)
6	J. H. Moon, R. Nakata, et al.	Human body model Subjects were provided with a diet containing onion slices (keratocortin equivalent to 67.6 to 93.6 mg/day)	Following brief consumption of quercetin-rich vegetables, conjugated quercetin metabolites accumulate specifically in human blood plasma, with concentrations ranging from 10(−7) to 10(−6) M.	(153)
7	Young J. Moon 1, Liang Wang, Robert DiCenzo, et al.	Human model Subjects received 500 mg of quercetin three times a day, and blood and urine samples were collected.	The oral clearance (CL/F) of quercetin was high (3.5 × 10(4)l/h) with an average terminal half-life of 3.5 h.	(149)
8	Navindra P. Seeram 1, William J. Aronson, Yanjun Zhang, Susanne M. Henning, et al.	C57BL/6 wild-type male mouse model A pomegranate extract (PE) abundant in ETs, which upon hydrolysis yield ellagic acid, was administered to male C57BL/6 wild-type mice. Metabolite concentrations in plasma and tissues were assessed within a 24 h timeframe.	Prostate, colon and intestinal tissue of the mice ET metabolites concentration is higher, further determine the metabolism and tissue distribution of ET metabolites	(180)
9	I-Chen Lin, Jin-Yi Wu, et al.	Mouse model Ellagic acid (1.2 mg/rat/day, *n* = 10)	Ellagic acid levels can be detected in fecal samples, but not in plasma or liver	(165)
10	Navindra P. Seeram, Rupo Lee, David Heber, et al.	Human body model A serving of 180 mL pomegranate juice comprised 25 mg of ellagic acid and 318 mg of ellagitannins compounds.	Maximal concentrations of ellagic acid (31.9 ng/mL) were detected in human plasma 1 h after digestion, but were quickly eliminated by 4 h	(168)
11	B. Cerdá, C. Soto et al.	Human body model Randomized, double-blind, placebo-controlled study, participants consumed either 400 mL of pomegranate juice with ellagic acid or a placebo daily over a 5-week period.	No polyphenols, including ellagic acid, from pomegranate juice were found in the plasma or urine of volunteers.	(170)
12	Jun-Hui Choi, Jong-Kook Park et al.	The cell model Ferulic acid (~ 300 μg/mL)	There was no obvious toxicity to the three cell lines of platelet, leukocyte and erythrocyte.	(181)

## 5 Conclusion

The purpose of this review has been to investigate the potential preventive effects of polyphenols on obesity, focusing on their effects on fat cells and their ability to regulate fat metabolism and intestinal flora and exploring their relationship with obesity-related signaling pathways from an obesity prevention perspective—for example, increasing BAT activity, increasing protein factors that regulate brown fat thermogenesis, increasing thermogenesis, regulating the Rb pathway, P38/MAPK pathway, Wnt signaling pathway, and transcription factor regulation to inhibit adipogenesis. The AMPK pathway regulates fat metabolism through lipase catalysis, while the PI3K/AKT signaling pathway alleviates insulin resistance, activates the AMPK signaling pathway, directly inhibits inflammatory factors, thereby inhibiting inflammation, and regulates the gut by improving the composition, proportion, and diversity of intestinal microbes. It should be noted that polyphenols’ therapeutic effect on obesity is limited, and further research and extensive clinical trials are required to treat obesity with polyphenols.

The main challenge in this research field is polyphenols’ low bioavailability. Many representative polyphenols and their analogues do not fully possess the properties necessary for ideal drug candidates, which include insufficient chemical stability, low water solubility, low efficacy and selectivity for target action, poor distribution in multiple tissues, unstable metabolic processes, and relatively high toxicity. In the future, it may be possible to improve polyphenols’ bioavailability of polyphenols through lipid vesicles, nanoparticles and nanofibers, chemical modification, polymer micelles, and chitosan nanoparticles (131).

## Glossary

BMI: body mass index
WAT: white adipose tissue
BAT: brown adipose tissue
UCP1: uncoupling protein 1
NF-κB: nuclear factor kappa-B
IL-6: interleukin-6
LPS: Lipopolysaccharides
CD14: cluster of differentiation 14
BAs: bile acids
HFD: high-fat diet
GM: Gut Microbes
PGC1α: Peroxisome Proliferator-Activated Receptor Gamma Coactivator 1-α
CPT1: carnitine palmitoyltransferase 1
Rb: Retinoblastoma
P38 MAPK: P38 mitogen-activated protein kinase
PPARγ: Peroxisome Proliferator-Activated Receptor γ
CEBP-β: CCAAT Enhancer-Binding Protein β
IL-1β: Interleukin-1β
IEC: intestinal epithelial cells
Wnt: Wingless/Integrated
CEBP-α: CCAT/enhancer-binding protein α
TRAF4: TNF receptor-associated factor 4
CARM1: histone arginine methyltransferase 4
AMPK: AMP-activated protein kinase
ACC2: Acetyl-CoA carboxylase 2
STAT3: transcriptional activator 3
FOXO1: Forkhead box O1
GLUT4: Glucose Transporter 4
TG: Triglyceride
HO-1: Heme Oxygenase-1
TNF-α: Tumor Necrosis Factor-alpha
LFD: low-fat diet
